# Exploring the psychological and religious perspectives of cancer patients and their future financial planning: a Q-methodological approach

**DOI:** 10.1186/s12904-022-01079-z

**Published:** 2022-10-18

**Authors:** Kanwal Iqbal Khan, Qurat ul An Sabir, Ambreen Shafqat, Muhammad Aslam

**Affiliations:** 1 Management Sciences Department, New Campus, University of Engineering and Technology, Kala Shah Kaku, Pakistan; 2 School of Statistics, Minhaj University Lahore, Lahore, Pakistan; 3 Biofluids and Biosystems Modelling Lab (BBML), Department of Engineering, Faculty of Agriculture, Dalhousie University, Nova, Scotia Canada; 4 Department of Basic Sciences, School of Mathematics and Statistics, Riphah International University, Islamabad, Pakistan; 5 Department of Statistics, Faculty of Science, King Abdulaziz University, 21551 Jeddah, Saudi Arabia

**Keywords:** Cancer Patients, Q-methodology, Religious beliefs, Psychological impacts, Financial Burden, Personal and Financial Planning, Fear and Acceptance of Death

## Abstract

**Background:**

Cancer patients are often hesitant to talk about their mental health, religious beliefs regarding the disease, and financial issues that drain them physically and psychologically. But there is a need to break this taboo to understand the perceptions and behaviours of the patients. Previous studies identified many psychological factors that are bothering cancer patients. However, it still requires exploring new elements affecting their mental and physical health and introducing new coping strategies to address patients’ concerns.

**Methods:**

The current study aims to identify cancer patients’ perceived attitudes towards the severity of illness, understand their fears, tend towards religion to overcome the disease, and future financial planning by using a Q-methodological approach. Data were collected in three steps from January-June 2020, and 51 cancer patients participated in the final stage of Q-sorting.

**Results:**

The findings of the study are based on the principal component factor analysis that highlighted three essential factors: (1) feelings, (2) religious beliefs about the acceptance of death, and (3) their future personal and financial planning. Further, the analysis shows that the patients differ in their beliefs, causes and support that they received as a coping mechanism.

**Conclusion:**

This study explains cancer patients’ psychological discomfort and physical pain but cannot relate it to co-morbidities. Q methodology allows the contextualization of their thoughts and future planning in different sets, like acceptance of death, combating religion’s help, and sharing experiences through various platforms. This study will help health professionals derive new coping strategies for treating patients and financial managers to design insurance policies that help them to share their financial burdens.

## Background

Cancer is the uncontrolled growth of abnormal body cells. Its diagnosis affects not only the physical condition of patients but also emotionally drains their families. It is a life-changing experience. Depression and anxiety are the most common side effects [1, 2]. The whole life turns upwards down, and it is crucial to identify those changes and provide needed help [3]. The persons experiencing cancer not only bear the physical pain of surgery, chemotherapy, radiation, bone marrow transplant, and immunotherapy but also pass through psychological trauma that can badly affect their physical and mental health [4, 5]. Patients considered themselves a burden to their family and friends, often resulting in self-harm and suicidal thoughts [6, 7]. The interpersonal-psychological theory of attempted and completed suicide also regarded a sense that considering oneself as a burden on others is one of the essential components of ending their life by suicide [8].

Psychology and its theories help us to understand patients’ behaviour [9]. Behavioural sciences theories describe the feelings of individuals and how they define and interpret disease. It also explains their acceptance and fears of death, future planning, and remedial actions towards it. These factors are shaped by sociocultural and psychological behaviour rather than cognitive, physiological, genetic, or other biological reasoning for the disease [10]. Thus, illness behaviour reflects complex reactions toward changing bodily sensations that represent the psychological predisposition of the person and the broader socio-economic context within which the individual lives [11].

Although previous studies reported many psychological problems, confronting cancer patients such as thread to life, anxiety, body image concerns, financial crisis, increased marital stress, fear of being unemployed, not capable of fulfilling the social roles in life etc. that badly impact their mental and physical health [9]. But still, there is a need to study the perceptions and behaviour of cancer patients to explore some new factors that are bothering them. Much research is conducted to analyze the reactions of cancer patients towards the severity of their disease and their co-existing worries about adverse psychological long-term consequences of treatment [12]. These factors also slow down their recovery process and become a hurdle to obtaining the desired results.

Health practitioners often use psychological theories to design coping strategies for cancer patients. Bandura’s self-efficacy theory helped to develop an effective psychological treatment framework for cancer patients [13]. These treatment strategies are useful in dealing with the emotional distress of the patients through psychological intervention. The social cognitive theory provides a support mechanism that improves patients’ overall quality of life [9]. Religious beliefs and spirituality also play a significant role in the treatment process by creating a ray of light among the patients that positively impacts their lives [14]. Religious beliefs act as a coping-up strategy that supports the illness and positively deals with it [15, 16]. It serves as a long-term therapy that results in maintaining the self-esteem of patients, restoring their confidence, giving them emotional comfort, and creating a sense of meaning in life [17, 18].

Family and social support are also considered essential for the psychological well-being of the patients. However, the finest moral and psychosocial support demands understanding individual and family-level perceptions at the time of cancer diagnosis and throughout the treatment trajectory [19]. The patient’s willpower and spiritual therapy play a vital role in cancer treatment [20]. Previously most of the research involved the caretakers asking about the patient’s feelings which did not directly depict the feelings of patients [4, 8]. The current study targeted cancer patients and directly explored their feelings and opinions.

Similarly, positive patient-doctor communications provide undue support to patients to come out of the trauma [21]. A positive patient-doctor relationship helps adaptation to illness, reduces treatment pain, and provides hope to fight against the disease. The nursing interventions also support building an empathetic relationship with the patient and their family members that help in fostering mutual trust and facilitating coping mechanisms during the care process [22]. But patients with antisocial personality traits have more psychological order and face difficulty in handling it [23].

Finances are a big question mark for patients to bear the cost of treatment besides the psychological issues. Scholars believe that cancer treatment costs have a profound, long-lasting impact on the pockets of patients and caretakers [24]. Families often become indebted or bankrupt as they do not want to compromise their patients’ health and functional outcomes [25, 26]. So, financial issues are considered the highest risk factor in psychosocial oncology for patients and their families during treatment [27]. Patients are also worried about the future of their families. They must re-evaluate their priorities and take strict actions for their family security. Previous studies focused on the bidirectional impact of family-reported positive (resilience) or negative (distress) psychosocial well-being. Still, none have explicitly focused on the patient’s feelings, fears and coping strategies and particularly their future financial planning to secure their family’s future [3].

The study aims to identify cancer patients’ perceived attitudes towards the disease severity and understand their fears and future financial planning. Previous researchers explored various psychological issues, religiosity and spirituality factors and the economic burden of the disease either through qualitative methods or quantitative techniques. However, the current study explores the perceived feelings of cancer patients on these issues jointly by using the Q-methodological technique, a combination of qualitative and quantitative approaches. In this way, it will provide a comprehensive view of the patients and further contribute to the current knowledge in psychology, oncology, and behavioural sciences studies.

## Method

This study aims to identify the perceived attitude of cancer patients towards their disease by applying a Q-methodological approach and describing their resultant actions regarding future planning. Q methodology is a novel approach and gives the foundation for the analytical study of people’s opinions, attitudes, feelings and viewpoints [28]. It combines qualitative and quantitative techniques that depict a comprehensive viewpoint of the respondents [29].

### Data collection procedure

Data were collected in three steps. In step 1, a Q sort pack of statements was developed through literature review, asking a global single-item question from the relevant stakeholders and in-depth interviews. The second step involves finalizing the Q-sample statements from Q-population based on the expert’s opinions in the field. In the last step, questionnaire items were finalized by the experts, and data were collected from the final respondents. Data was taken from cancer patients in Pakistan from January-June 2020.

### Construction of concourse (Q population)

The first step in Q methodology is to develop the Q sort pack, preferably a set of 40 to 80 statements relating to the topic of study [30]. Q-concourse of statements were developed through Global Single-Item Questions and in-depth interviews. The initial Q-concourse (collection of opinion statements to represent possible reactions towards the severity of disease) was assembled after reviewing relevant literature [31, 32]. The interviews and written narratives are based on the Global Single-Item Question: “what are the feelings of cancer patients towards illness and their future planning?” The question was asked from 31 adults, 12 immediate family members of cancer patients, 5 oncologists, 10 caretaking nurses, 3 psychologists and 2 general physicians, who were not the study participants.

Further, 5 in-depth interviews were conducted with cancer patients. These interviews unveil their feelings, reactions, and experiences about the disease, their journey from fear to acceptance of death and their future planning (domestic, financial, and personal satisfaction to the soul). Finally, we ended up with a total of 121 statements as a Q population. We carefully selected samples by keeping the margin of error (confidence interval) by +/- 5%. We chose a confidence level of 95%, and variability (standard deviation) among the sample was 0.5, and we calculated a sample size of 51 using Eq. (1)

$$Necessary\,Sample\,Size\, = \,N\, = \,{Z^2}\,.\,\sigma \,(1\, - \,\sigma )/{e^2}$$ (1)

*where e = margin of error*.

### Q-sample

In the second step, the Q sample was finalized, a set of selected statements from the Q population based on the experts’ opinions in the field. The experts (4 professors and one methodologist) analyzed 121 statements and rank-ordered them according to their meanings and context. They ended up with 46 statements as a Q sample, divided into 3 main categories: feelings of cancer patients (17), religious beliefs about the acceptance of death (16), and future personal and financial planning (13). This sample is based on the most representative and distinctive statements that are considered best for use in the Q sorting process.

### Selection of participants

In the third step, the study participants were selected who were cancer patients admitted or taking treatment from the local cancer hospitals in Pakistan. This study is conducted keeping in view the cultural and social norms of Pakistani society. The health system is entirely different here. The government and private sectors provide no health insurance. Chronic diseases like cancer may dig a hole in the pocket of the common person, which affects their emotional and financial state. The family also suffers a lot, and depression is quite common in this scenario. An essential advantage of Q-methodology is using a small sample of purposively selected respondents, which is more helpful in predicting intra-individual differences rather than inter-individual [25]. Therefore, a sample of 60 participants was employed based on their agreement to contribute to this study. Further, participants were ensured that the provided information would be used anonymously for research purposes only. Researchers maintained a high level of confidentially during the study’s complete process. Nine participants withdrew because they were too demanding (2); had changed their mind (4); were not comfortable (1); or were so tired (2). Finally, 51 participants (85%) attended and completed the Q sorting process.

### Q sorting

The researchers have done multiple meetings with the study participants who were agree to participate. During the initial meetings, we elaborated the objective of study, how they can contribute to our study, listened to their concerns and those who gave their verbal consent to we took data from them. The data were collected in two stages. In stage I, 51 study participants answered the survey with 46 Q statements on a likert scale of 1 (strongly disagree) to 7 (strongly agree). These statements were finalized after following a approved process mentioned in the paper. In stage 2, respondents were asked to explain the preferences which they made in the survey to make sure they fully understand the concept of study. What is the reason behind their choices?. The researchers have taken all the notes and tried to provide an easy and convenient environment for them as they were going through an emotional phase. All these responses are reported in the results section. However, the resultant Q sorts representing the participant’s operant subjectivity on the issue under consideration are presented.

## Results

The sample characteristics are shown in Table 1, which varied within the groups. Of the 51 respondents, 55% were female, 57% were married, and the dominant age group was 36 to 45 (25%). Breast cancer is the most prevalent type of cancer among the study participants. However, all cancer types mentioned in the table prevail in Pakistan [31]. But, the number of patients with breast cancer is the highest of all [33]. Most of the participants are employed (51%) and have completed at least their college education (47%). Further, the characteristics of the respondents are shown in Table 1.

**Table 1:** Patients Demographics

Cancer Patients (n = 51)	n (%)
**Gender**	
Male	23 (45)
Female	28 (55)
**Marital Status**	
Single	14 (27)
Married	29 (57)
Separated/Divorced	3 (06)
Widow	5 (10)
**Age**	
15–25	10 (20)
26–35	12 (24)
36–45	13 (25)
46+	16 (31)
**Education**	
High School or Less	7 (14)
College or more	24 (47)
University or more	20 (39)
**Employment Status**	
Employed	19 (51)
Unemployed	15 (41)
Retired	3 (08)
**Cancer Type**	
Breast	14 (27)
Lip, oral cavity	10 (20)
Lung	7 (14)
Oesophagus	5 (10)
Leukaemia	4 (08)
Cervix uteri	4 (08)
Ovary	5 (10)
Other	2 (04)

The study is exploratory, so there is a need to assess the validity of the data. Most Q-methodology studies are exploratory and qualitative and tend not to use random sample designs. That is why questions of the research validity were assessed differently from quantitative research methods [34, 35]. As understood in more conventional survey research, item validity does not apply to the study of subjectivity. In Q-methodology, one expects the meaning of an item to be interpreted individually. The contextual meaning of how each item was individually interpreted becomes apparent in the rank-ordering and follow-up interviews.

It shows the factor characteristics explaining the average reliability coefficient used to assess the reliability, or internal consistency, of a set of scale or test items. In other words, the reliability of any given measurement refers to the extent to which it is a consistent measure of a concept. Cronbach’s alpha is one way of measuring the strength of that consistency. Due to this reason, the appropriate statistical techniques are used to achieve the objectives of the study. Reliability analysis was done to check the quality of the survey, which is suggested as an estimate of reliability [36]. If the value of Cronbach’s alpha is between 0.60 and 0.90, data is considered highly reliable and consistent [36]. Our Cronbach’s alpha score is 0.774, which shows that the data is reliable and consistent.

Table 2 shows the results of summary statistics of the Q sort items in the form of mean, standard deviations and Z score values. We first rank all the statements based on Z scores in descending order and then rank them according to the mean and standard deviation values, respectively. All the sample statements were sub-categorized into three main factors presented in Table 2. It presents three significant factors about the fear and psyche that cancer patients recognize: psychological and emotional needs (17 statements), fear of death and dependency on religion (16 statements), and future financial planning (13 statements). We analyzed the data by multiple correspondence analysis (MCA), and all the noises from the data were removed to obtain good results.

**Table 2:** Summary Statistics

Item no.	Mean	Z-score
**Factor I: Feelings of Cancer Patients**		
**2**	4.04	2.21
**9**	3.24	2.15
**3**	4.16	2.14
**10**	3.25	2.06
**5**	3.1	2.05
**7**	3.65	2.03
**14**	3.63	2.03
**4**	4.18	1.94
**12**	3.96	1.90
**16**	4.10	1.89
**8**	3.59	1.89
**6**	3.67	1.88
**1**	4.06	1.82
**11**	3.45	1.76
**13**	3.16	1.71
**15**	3.20	1.67
**17**	3.84	1.41
**Factor II: Religious Beliefs about the Acceptance of Death**		
**30**	3.24	2.10
**20**	3.37	2.02
**27**	2.98	1.91
**32**	5.25	1.90
**24**	3.18	1.90
**28**	2.57	1.82
**21**	3.53	1.81
**22**	3.39	1.80
**25**	3.18	1.80
**29**	2.78	1.74
**23**	3.25	1.71
**19**	3.55	1.70
**31**	5.71	1.69
**26**	2.78	1.68
**18**	3.71	1.62
**33**	5.41	1.51
**Factors III: Future Personal and Financial Planning**		
**44**	4.57	1.98
**38**	5.00	1.80
**35**	4.98	1.79
**37**	4.78	1.78
**43**	4.63	1.75
**34**	5.02	1.71
**42**	5.04	1.60
**36**	5.29	1.59
**45**	5.24	1.56
**40**	5.18	1.56
**46**	5.65	1.51
**39**	5.43	1.47
**41**	5.24	1.45

MCA consequently played an essential role in data screening, so our selected Q-factors are simpler and more accurate. Applying MCA to data, Table 3 shows that total inertia is 0.79 (percent of inertia 45% is due to the first axis & 34% is due to the second axis). Total inertia values indicate how much variability is in the model. Each dimension’s inertia values refer to the amount of variance by each dimension [34]. We have selected the highest interaction factors and ignored the weak relationship factors through MCA. Data were collected from 51 participants to check cancer patients’ views on how they are combating their disease, e.g. by improving their mental health with the help of religion and if they have any financial planning. Cochran’s Q test determined a statistical significance in the proportion of patients coping with their disease by different means over the time χ^2^(2) = 493.46, *p* < .05, see Table 4.

**Table 3:** Impact of All Variables

Model Summary			
**Dimension**	**Cronbach’s Alpha**	**Variance Accounted For**	
		Eigenvalue	Inertia
**1**	0.95	5.819	0.45
**2**	0.94	3.763	0.34
**Total**		9.582	0.79

**Table 4:** Cochran’s Q Test

		Sum of Squares	df	Mean Square	Cochran’s Q	Sig
**Between Factors**		672.9	50	13.45		
**Within Factors**	**Between factors**	1877.8	45	41.73	493.45	0.001
	**Residual**	6855.6	2250	3.047		
	**Total**	8733.5	2295	3.805		

The critical statements from each of the three factors were sorted through PQ method 2.11 (statistical method: Multiple correspondence analysis to select the high interaction terms), which gives us the dimensions and insight of the Eigenvalues; we selected our Q factors based on these insights. The most acceptable factors were decided based on Eigenvalues which are at least 1.0. We have rearranged the selected Q-sorts based on Z scores in Table 5. The resultant factors are divided into three main categories: feelings of cancer patients, religious beliefs about accepting death, and future personal and financial planning.

**Table 5:** Descending Array of Z-scores Presenting Feelings of Cancer Patients towards Illness and Their Future Planning

Item No.	Statements	Z-score
**Factor I: Feelings of Cancer Patients**		
**9**	I was not mentally ready for all this	2.15
**8**	When this news broke, I was in a state of shock and disbelief and felt numb.	2.03
**14**	I often think, why me? Why did God let that happen to me?	2.02
**20**	I am worried about the cost of treatment.	2.02
**12**	Thoughts came to my mind that people feel pity and grief when they came to know about my disease.	1.90
**7**	I started getting panic attacks when the painful treatment process came to my mind.	1.88
**Factor II: Religious Beliefs about the Acceptance of Death**		
**27**	A person’s body will die but not the spirit.	1.91
**24**	Death is inevitable, so we should not worry about it	1.90
**21**	We should not think about death; we have to live fully and enjoy every moment of life	1.81
**31**	Social and family support lowers feelings of anxiety and depression.	1.69
**26**	Only religion can help a person overcome the fear of death and console the mind and body.	1.68
**33**	My willpower is giving me the strength to combat the disease.	1.51
**Factor III: Future Personal and Financial Planning**		
**44**	I will donate my organs (eyeballs, cornea, heart, kidney, etc.) to other people.	1.98
**38**	I will purchase investment plans for my family.	1.80
**35**	I will write a will regarding the distribution of my assets and unfulfilled wishes.	1.79
**34**	This disease has changed my retirement, travelling, or parenthood plans.	1.71
**19**	I am worried that I am causing trouble for my family and friends (emotionally and financially).	1.70
**36**	I will clearly instruct my family regarding my social responsibilities.	1.59
**45**	I will add a specific portion of my wealth to a charitable institution.	1.56
**40**	I will make diversified investments to minimize risk.	1.56
**39**	I prefer risk-free investments to secure my family’s future.	1.47
**41**	I will take the consultancy from financial experts (brokers, fund managers, bankers, and real estate agents) before finalizing my investment plans	1.45

### Feelings of cancer patients

Cancer patients in this factor appear in a challenging situation. Table 5 of statements where they mainly were strongly agreed or agreed. They were in a big shock and disturbed psychologically over the fact of why God had chosen them for this disease. According to their statements, they were distraught when this news was revealed. Results showed the perceived feelings of cancer patients; when they first received the news, they were in a state of shock. They felt panic and started questioning God, “why has he selected them for this disease? Why cannot he go for any other person”. Statistical results are significant about their feelings that they start feeling pity and jealousy from other people. Some people reported increased anxiety and panic attacks and started feeling depressed about their finances. A patient said, “when I received the news that I have cancer, I was shocked and could not utter a single word for some moments”. Feelings are different gender-wise; women were more emotional than men and were more composed.

### Religious beliefs about the acceptance of death

From Factor II, the most realistic statement is identified by the respondent that their belief in death is a certain thing. We all believe in that, but untimely or when you know about the time of your death, you feel pretty anxious, distressed, etc. This situation is more harrowing that counting the death at your fingertips. Participants classified their death-related thoughts, acceptance of death, and how religion helped them overcome this fear. Elderly patients believed in religion’s comfort; they stated that religion helped them a lot to fight with this fear, and God is gracious, and he will ease their pain. Old-aged persons had an increased tendency towards religion than young ones. In the light of the results, people believed in the certainty of death in the light of religion. A patient said, “He was not religious before, but after the disease, he started following the religion and that change helped him cope with the pressure of disease”.

### Future personal and financial planning

Factor III highlights the intensity of the respondents towards future financial planning. Cancer patients are already bearing the high cost of treatment, and patients, particularly older ones, are worried about their family’s future and want to secure it. They emphasized future financial planning for them and their families. Few participants wished to donate their organs after death to help humanity. They were worried about the cost of treatment because cancer treatment is costly. Z scores explained that patients felt a burden to family and friends. Some patients said, “They contacted the financial institutes for their future financial policies but found any suitable plan”. Some people wanted to donate their property to charitable institutes. The patients started planning the future of their families. Young people are more optimistic about their future, planning that they will recover soon and take a fresh start in their life. Some participants wanted to buy the investment plans and write the will for their families.

## Discussion

This study explored cancer patients’ behaviours and attitudes towards their death and future financial planning. It employs Q-methodology, which helps to identify the conflicting priorities of patients. The findings explain three main factors. Firstly, they feel financially drained over the cost of treatment because these treatments dig a hole in patients’ pockets. So there should be enough financial policies to help them and their families after death. The second difference in beliefs was noted about illness and death. Most people found that religion is helping them with medicine to cope with the disease. They believed God commands their lives, and everything happens according to his will. However, some did not but were willing to accept it and have different views. They preferred self-management as well as accepting medical treatments. Thirdly, some patients differed on the importance of supporting networks and not feeling shame in seeking help, which appeared we could protect them from suicidal thoughts or feelings.

In comparison, some patients felt unsupported and embarrassed and had to consider suicide to stop the distress [10]. Identifying depression in patients is crucial, and one should introduce the detection and treatment strategies in primary & aftercare. The patients emphasize the need to make those policies according to their personalized needs so they can recover physically and emotionally. Cancer patients have started feeling self-pity and burden on their families. They caught themselves in their thoughts about why this disease came to them, their lives changed upwards, and their energies were low in panic attacks. These thoughts are alarming because they reflect the psychological state of a cancer patient and how they are mentally disturbed when they become aware of their disease. It further highlights the need for a psycho-oncologist to handle cancer patients’ emotions and save them from depression and negative thoughts.

If we see the religious factor, some of the patients quoted that their painful thoughts often lead them to self-harm or have suicidal thoughts to ease the pain. Certain patients were hopeful for their life. They wanted to enjoy every bit of their life even though they were going to die [37]. They quoted that death is the new beginning, so there is no need to be frightened of it; on the other side, some feel comfortable with their families, which helps them ease the pain [20, 38]. A few relations stand out based on the data obtained from the questionnaire the respondents completed. They also had attitudes in common. Most of the elderly patients agreed with the statements about dying. A person dying should be given a chance to talk openly about their death and their psychological and physical needs to their families and doctors without being judgemental [39–41] because the pain of unfulfilled things and wishes can be seen in their eyes.

Regarding future planning, patients appeared to be in a difficult situation; some participants wanted to donate their wealth to charity because they believed it would soothe their souls and help them even after death [37]. Some participants wanted to buy the investment plans and write the will for their families. But few of them had clear goals. They want to address their family so that they will know about their future unfinished work, social responsibilities, and hidden personality traits. This thought usually prevails among young dying patients who have kids. They wanted to nourish the psychological needs of their growing-up kids by doing so.

### Study implications

The current study benefits the scholars, psychologists, oncologists and managers in multiple ways. Firstly, it will help the families of cancer patients to understand and cope with the feelings of their suffering loved ones. Secondly, it will be beneficial to understand the psyche of cancer patients and observe the changes in their behaviour and uncertainty about future accomplishments during the painful process of treatment that affects their daily activities. Thirdly, it will help the oncologists and psychologists work in a team to plan medication with counselling services for cancer patients and implement treatment plans more effectively. Depression remains highly predominant in cancer patients and dramatically impacts their quality of life; perhaps utilizing its impact on observance, physical activity, social support, etc., will highlight the need to address the new health policies. Psychologists and oncologists can make new policies with their mutual discussion with the help of this study. Fourthly, it will help financial institutions to deal with the mortality fears of cancer patients and design their policies in light of the study’s findings.

### Study limitations

Some limitations should be used to evaluate this study correctly. First, it discusses psychological discomfort and physical pain. Still, we cannot find its relation with the co-morbidities effect, which is a new research direction for future studies to investigate the appropriate psychosocial care for cancer patients. Second, data were collected from a single country, where cultural and socio-economic conditions are diversified from other countries. Third, the study’s religious beliefs, family backgrounds, and social responsibilities may also vary, influencing the findings little. Also, when this data was collected, a pandemic in the form of Covid-19 had hit Pakistan, and due to this pandemic, people’s beliefs and thoughts changed, and they turned towards religion more than ever. That is because cancer patients were away from their loved ones and not allowed to meet them due to the SOPs followed by the hospital’s administration to keep them safe from COVID-19 has impacted the patients. They felt lonely in those hospital beds and found relief in religion and coping with the disease during those hard times with their disease [42]. Fourthly, we have used the minimum sample size that fulfils all the properties of the excellent estimator for selecting the sample size, and it works well with the Q methodology.

## Conclusion

This study is conducted to identify cancer patients’ perceived behaviour towards their disease using the Q-methodological technique. The participants shared their experiences with illness, including psychological distress, fear of dying, concerns about treatment cost, future uncertainties, and combating it. The findings reported three key factors: feelings of the cancer patients, their religious and spiritual beliefs, and future personal and financial planning. Their responses also varied according to age, gender, disease severity, and recovery expectations. Young people are more enthusiastic about their future, while older ones, particularly cancer patients of stages III and IV, are pretty uncertain about their lives.

Results showed that they all face a specific degree of stress and anxiety when they know about their disease, and it was difficult for them to accept this reality initially. But their religious beliefs, social support, and health practitioners play a positive role in their lives, keeping them hopeful and serving as a coping-up strategy. Young people, who are married and have family responsibilities, face more financial distress, like fear of losing jobs. Married women were more worried about their kids. The patients also discussed their future personal and financial planning. The present study will help practitioners to improve their treatment strategies, and design customized plans according to patients’ needs and behaviours. It will help to create a trusted atmosphere which will improve their mental health, peace of mind, and physical health.

## Data Availability

The datasets used and/or analyzed during the current study are available from the corresponding author upon reasonable request.

## Declarations

### Relevant guidelines and regulations

We confirm that all methods were carried out under relevant guidelines and regulations.
